# Molecular characterization of a novel ring 6 chromosome using next generation sequencing

**DOI:** 10.1186/s13039-016-0245-9

**Published:** 2016-04-21

**Authors:** Rui Zhang, Xuan Chen, Peiling Li, Xiumin Lu, Yu Liu, Yan Li, Liang Zhang, Mengnan Xu, David S. Cram

**Affiliations:** Center for Obstetrics and Prenatal Diagnosis, The Second Affiliated Hospital of Harbin Medical University, 150000 Harbin, China; Department of Obstetrics and Gynaecology, The Second Affiliated Hospital of Harbin Medical University, 150000 Harbin, China; Translational Medicine Center, Guangdong Women and Children’s Hospital, Guangzhou, 511400 China; Berry Genomics Corporation, Building 9, No 6 Court Jingshun East Road, Chaoyang District, Beijing, 100015 China

**Keywords:** Karyotyping, Ring chromosome, Chromosome microarray analysis, Copy number variation, Next generation sequencing

## Abstract

**Background:**

Karyotyping is the gold standard cytogenetic method for detection of ring chromosomes. In this study we report the molecular characterization of a novel ring 6 (r6) chromosome in a six-year-old girl with severe mental retardation, congenital heart disease and craniofacial abnormalities.

**Methods:**

Cytogenetic analysis was performed by conventional karyotyping. Molecular genetic analyses were performed using high-resolution chromosome microarray analysis (CMA) and next generation sequencing (NGS). OMIM, UCSC and PubMed were used as reference databases to determine potential genotype to phenotype associations.

**Results:**

Peripheral blood and skin fibroblast karyotyping revealed the presence of a dominant cell line, 46,XX,(r6)(p25.3;q27) and a minor cell line 45,XX,-6. Molecular karyotyping using NGS identified 6p25.3 and 6q27 subtelomeric deletions of 1.78 Mb and a 0.56 Mb, respectively. Based on the known genes located within the r6 deletion interval 6q25.3-pter, genotype to phenotype association studies found compelling evidence to suggest that hemizygous expression of disease genes *FOXC1, FOXF2, IRF4* and *GMDS* was the main underlying cause of the patient’s phenotype. We further speculate that the severity of the patient’s symptoms may have been exacerbated by low-level instability of the r6 chromosome.

**Conclusion:**

This is the first report of a novel r6 chromosome characterized at the molecular level using NGS.

## Background

Human ring chromosomes were first reported in 1956 from cytogenetic analyses of tumor cells [1]. Ring chromosomes can involve any of the 24 chromosomes and are recognized in approximately 1 in 25,000 conceptions by karyotyping [2]. Random de novo recombination events during gametogenesis or early pre-implantation embryo development are believed to be responsible for the formation of ring chromosomes. The most common mechanism of ring chromosome formation usually either involves breakage near the termini of the short and long arms or breakage of one of the arms, followed by a subsequent fusion to generate a circular shortened chromosome with two or one terminal deletions [3, 4]. Occasionally, telomere-telomere fusions can also occur without the loss of any significant chromosomal material to generate complete ring chromosomes [3, 5, 6]. In general, the overall phenotype of the patients with ring chromosomes are highly variable, but generally overlap with phenotypes of known chromosome disease syndromes associated with similar interstitial copy number variations (CNVs) [4].

De novo ring chromosome 6 (r6) is a rarely observed structural abnormality compared to other types of ring chromosomes, with just over 30 case reports published in the literature [7]. Several r6 cases have been serendipitously detected by karyotyping during routine prenatal diagnosis [8, 9]; however, the majority of cases have been revealed postnatally following genetic investigation of children with unexplained clinical features. The most common r6 variants reported involve terminal 6p deletions extending to p25 or p24 in addition to 6q deletions extending to q26 or q27 [10–15]. In a review of selected r6 cases [7], phenotypes were highly variable, with the most consistent clinical features involving mental and developmental retardation, in association with facial dysmorphic features including microcephaly, microgathia, short neck, flat or broad nasal bridge, epicanthus bilateral and malformations of the ocular and auditory systems.

Apart from karyotyping and fluorescent in situ hybridization with 6p and 6q probes, most reported cases pre-date the availability of high resolution molecular techniques such as array comparative genomic hybridization (CGH) and next generation sequencing (NGS) and thus determination of precise genotype to phenotype associations has been limited. Recently, by applying an NGS method called copy number variation sequencing (CNV-Seq), we were able to identify the precise terminal deletion intervals in three cases of r14, r22 and r18 chromosomes [16]. In this study, using both array CGH and CNV-Seq, we report a comprehensive molecular characterization of a novel r6 chromosome in a six-year-old girl with severe intellectual disability, congenital heart disease and dysmorphic craniofacial features.

## Results

### Clinical evaluation of the patient

A 31-year-old father and mother presented at our prenatal diagnostic department for clinical assessment of their six-year-old daughter with unexplained dysmorphic facial features and severe intellectual disability. In review of the family, both parents were healthy and there was no history of genetic diseases or birth defects. Their daughter was born at the gestational age of 40 weeks via emergency caesarian section with a low birth weight of 2200 grams.

**Fig. 1:**
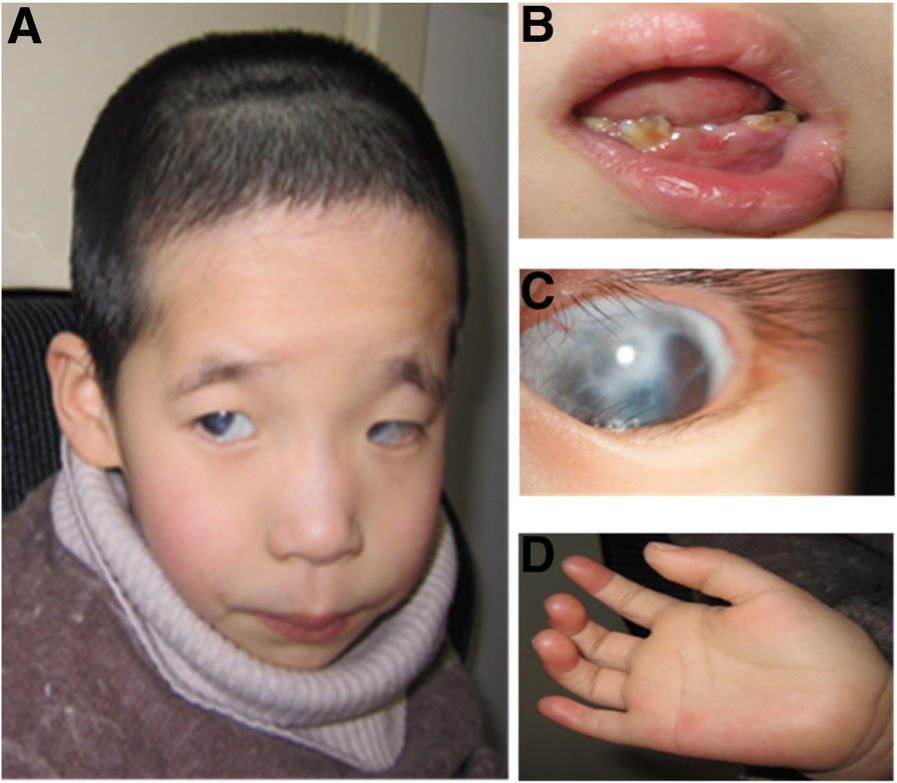
Facial features of the patient. Craniofacial abnormalities (**a**). Teeth agenesis (**b**). Ocular abnormalities (**c**). Shortened and (**d**). incurred small finger

When examined at the age of 6.5 years, her weight was 14.00 kg (< third percentile), length 103 cm (< third percentile), head circumference 42 cm and chest circumference 51 cm. A thorough physical examination revealed microcephaly, a low posterior hairline, dysmorphic facial features including microphthalmia, epicanthus, leukoma, nystagmus, iridogoniodysgenesis, a down slanting brow and canthus, a flat nasal bridge and tooth agenesis. She also had a short neck and flat occiput, widely spaced nipples, short and an inturned recurved little finger (Fig. 1). By two-dimensional color-doppler echocardiography, congenital heart disease was detected involving an ostium secundum defect (left to right shunt), patent ductus arteriosus (left to right shunt), pulmonary stenosis, left superior vena cava residues and coronary sinus distention. In addition, the girl exhibited developmental delay, mental retardation and speech difficulties, and her overall intellectual ability was judged to be equivalent to that of a one-year old infant. The girl was able to walk, but her gait was unsteady. She also presented with hyperactivity and gatism.

### Genetic investigation of the child

**Fig. 2:**
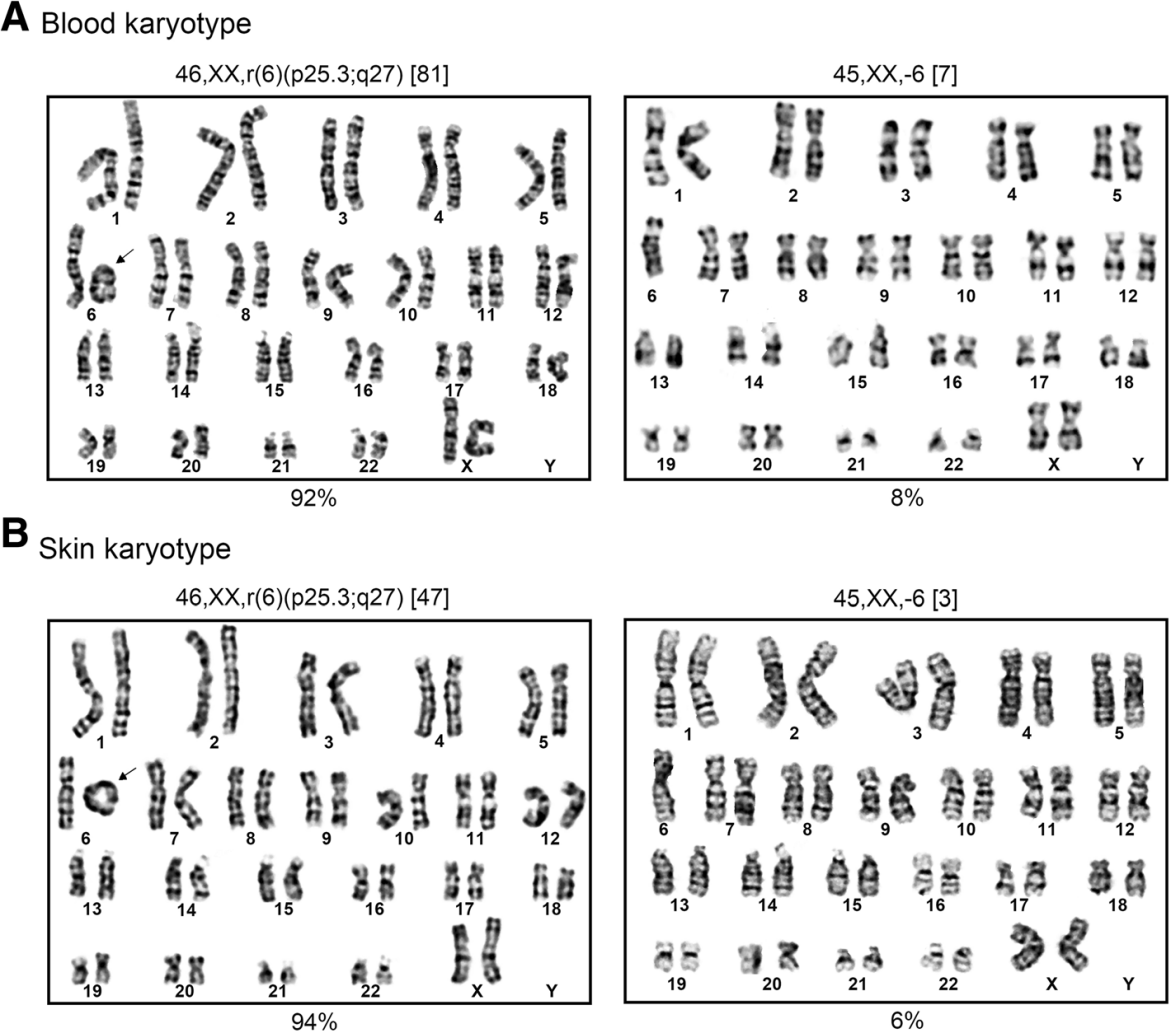
Tissue karyotyping. **a** Blood lymphocytes, 46,XX,r6(p25.3;q27)[81]/45,XX,-6[7]. **b** Skin fibroblasts, 46,XX,r6(p25.3;q27)[47]/45,XX,-6[3]. Loss of the r6 chromosome was seen in 8 % of blood lymphocytes and 6 % of skin fibroblasts

Conventional cytogenetic analysis of peripheral blood collected from the parents revealed normal karyotypes. Unexpectedly, their daughter returned a 46,XX,r(6) (p25.3q27) karyotype, involving one copy of a ring 6 chromosome and one copy of a normal chromosome 6. A further blood sample and a skin punch biopsy sample was collected from the patient to investigate the possibility of tissue mosaicism. From testing 88 lymphocytes, the karyotype was 46,XX,r(6)(p25.3;q27)[81]/45,XX,-6[7], revealing 8 % of cells without the r6 chromosome (Fig. 2a). Similarly, from testing 50 skin fibroblasts, the karyotype was 46,XX,r(6)(p25.3;q27)[47]/45,XX,-6[3] indicating that 6 % of cells had lost the r6 chromosome (Fig. 2b).

**Fig. 3:**
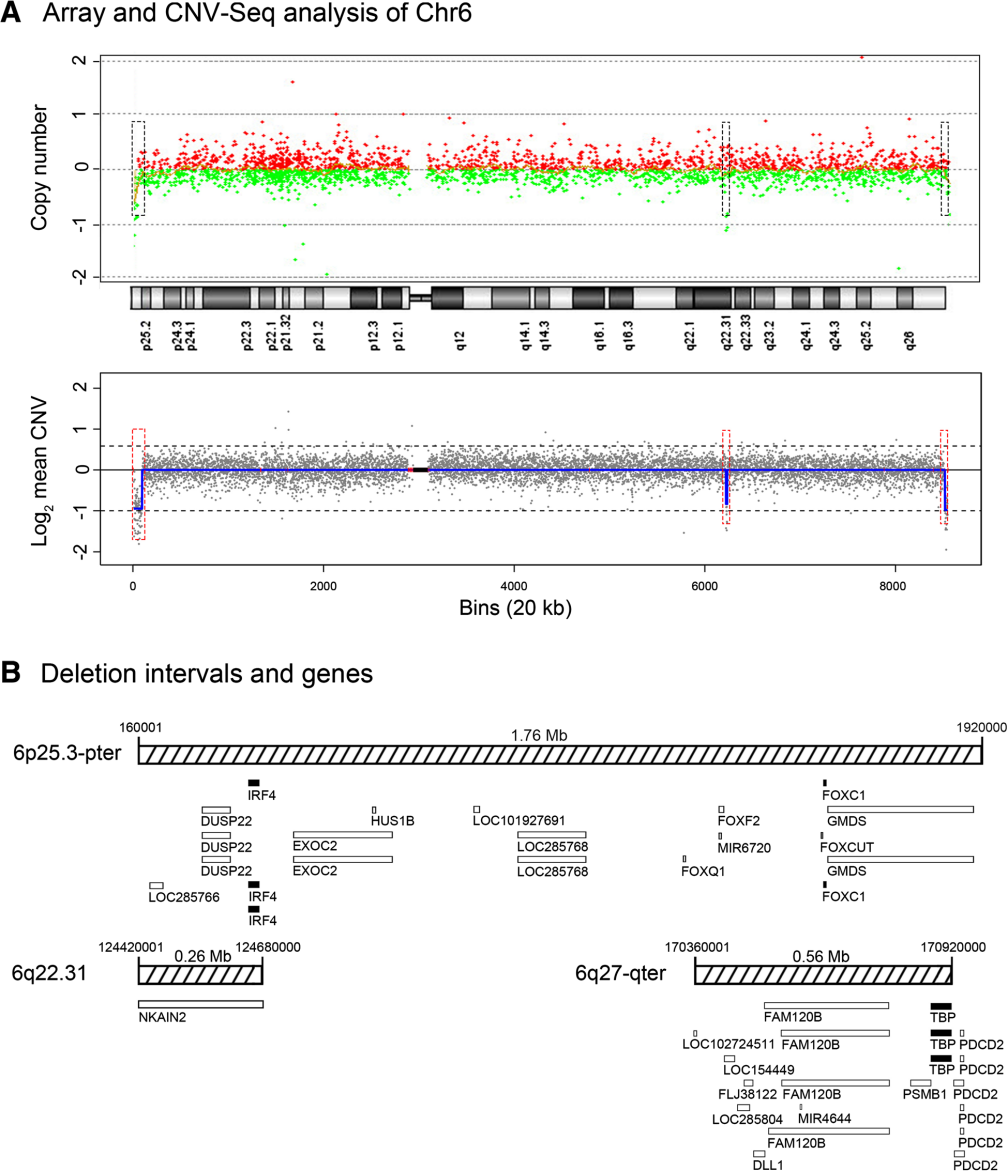
High-resolution analysis of r6 microdeletions and associated genes. **a** CMA and NGS copy number plots for chromosome 6. The array CGH plot is shown as copy number (Y-axis) versus cytogenetics co-ordinates. Red dots indicate chromosomal gain and green dots indicate chromosomal loss. The NGS chromosome 6 plot is shown as log_2_ mean CNV (Y-axis) versus 20 kb sequencing bins (X-axis). The blue line along chromosome 6 tracks the mean CNV. The upper dashed line represents a 100 % chromosome gain [log_2_(1.5)] and the lower dashed line represents a 100 % chromosome loss [log_2_(0.5)]. Red lines indicate regions of repetitive sequences and the black box marks the centromere. Three microdeletions were identified (red dashed boxes); 6p25.3-pter (detected by CMA and NGS), 6q22.31 (detected by NGS) and 6q27-qter (detected by NGS). **b** Gene deletion intervals. The relative size and position of UCSC database reference genes in the three intervals is shown. Open boxes represent non-disease genes and solid black boxes represent disease-genes, according to OMIM database

**Fig. 4:**
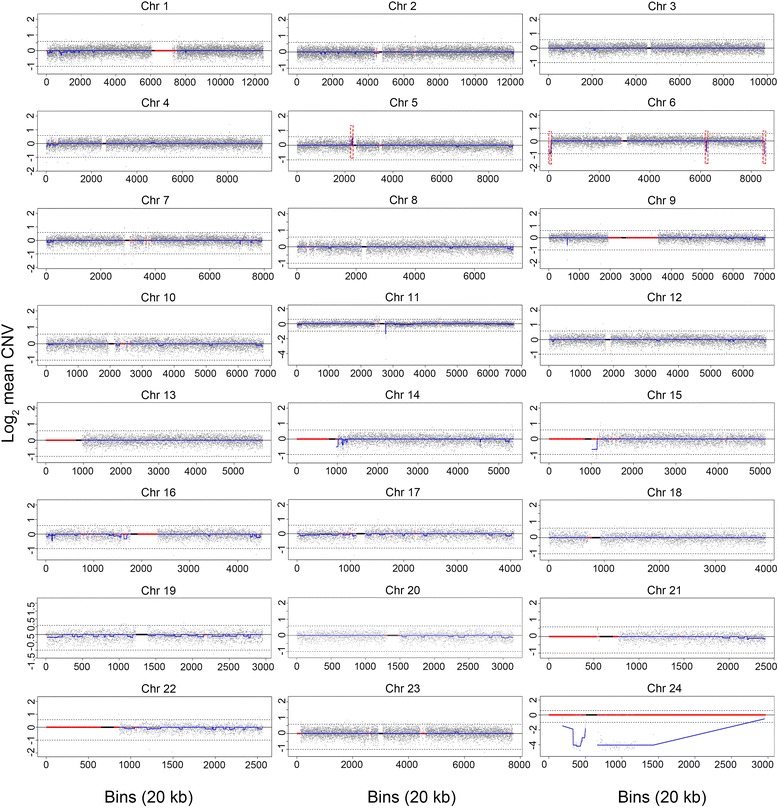
CNV-Seq analysis of patient genomic DNA. Twenty-four chromosome plots are shown as log_2_ mean CNV (Y-axis) versus 20kb sequencing bins (X-axis). Chr 23 = Chr X; Chr 24 = Chr Y. The blue line along the length of each chromosome tracks the mean CNV. The upper dashed line represents a 100 % chromosome gain [log_2_(1.5)] and the lower dashed line represents a 100 % chromosome loss [log_2_(0.5)]. Red lines indicate regions of repetitive sequences and the black box marks the centromere. Dashed red boxes indicate significant CNVs identified on chromosomes 5 and 6

To determine the molecular structure of the r6 chromosome in more detail, high-resolution array CGH was performed on genomic DNA extracted from peripheral blood (Fig. 3). Genome-wide profiling for pathogenic copy number changes by array CGH detected a 1.78 Mb 6p25.3-pter deletion. However, on chromosome 6, there was also evidence of a small intra-chromosomal deletion at 6q22.31 and a small terminal 6q27 deletion, respectively, although the “call” by the software was not confident due to the lack of informative probes in these two regions. To confirm the array CGH results, we also performed NGS using copy number variation sequencing (CNV-Seq). Sequence data analysis revealed four CNVs, namely a 0.66 Mb 5q11-12 duplication, a 1.78 Mb 6p25-pter deletion, a 0.26 Mb 6q22.31 deletion and a 0.56 Mb 6q27-qter deletion (Fig. 4).

Searches of the UCSC and OMIM databases for reference and disease genes were performed for the four CNVs defined by CNV-Seq to identify the genes encoded in these intervals (Fig. 3). Within the 6p25-pter deletion interval 13 reference genes were identified; namely, *LOC285766, DUSP22, IRF4, EXOC2, HUS1B, LOC101927691, LOC285768, FOXQ1, FOXF2, MIR6720, FOXCUT, FOXC1* and *GMDS,* where *IRF4* and *FOXC1* have been classified as OMIM disease genes. The 6q22.31 deletion interval encoded the gene *NKAIN2*, which has no known disease association. Within the 6q27-qter deletion interval there were 10 genes identified, namely; *LOC102724511, LOC154449, LOC285804, FLJ38122, DLL1, FAM120B, MIR4644, PSMB1, TBP* and *PDCD2,* where only *TBP* has been classified as an OMIM disease gene*.* The 5q11-12 duplication region contained no known genes. Literature searches found no evidence that this region is associated with pathogenicity, and on this basis, the 5q11-12 duplication was deemed to be benign.

## Discussion

This study presents detailed cytogenetic and molecular analyses to characterize a novel r6 chromosome originally detected by conventional karyotyping in a six-year-old girl. More extensive karyotyping of blood and skin cells showed that while the vast majority of cells had one copy of chromosome 6 and one copy of the r6 chromosome, a minority of cells in both tissues (6–8 %) had lost the r6 chromosome, resulting in monosomy 6. Since the blood karyotyping was performed on short-term cultures, and that long term cultures of skin fibroblasts did not increase the incidence of r6 chromosome loss, we conclude that the mosaic karyotype most likely originated in vivo due to r6 instability. High-resolution genomic analysis by array CGH and CNV-Seq was used to survey genome-wide CNVs and, simultaneously analyze the terminal CNVs associated with the r6 chromosome. The most significant CNVs identified were 1.76 Mb (6p) and 0.56 Mb (6q) subtelomeric deletions of the r6 chromosome. In comparison to other cytogenetically defined r6 chromosomes, this novel variant has the smallest 6p deletion involving p25.3-pter, whereas all other r6 variants reported to date have more extensive deletions, involving 25p-pter or 24p-pter [7].

By comparison, NGS provided a much higher resolution analysis of the patient’s DNA than array CGH, allowing precise definition of genome-wide CNVs and the subtelomeric CNVs associated with the r6 chromosome. While both NGS and array CGH identified the 1.76 Mb 6p subtelomeric deletion, array CGH missed the 0.56 Mb 6q subtelomeric deletion. In addition, NGS was able to accurately quantitate the copy number changes of the deletions. The difference in resolving power between the two techniques for the subtelomeric regions of chromosome 6 was attributable to the increased data points generated by NGS, which is based on analysis of randomly distributed sequencing reads whereas array CGH probes are more targeted to disease genes located throughout the genome. For example, to detect the 0.56 Mb 6q deletion, NGS utilized multiple data points provided by 28 contiguous 20 kb sequencing bins, which contain on average of 30–35 reads per bin [16]. In contrast, within this region, the array CGH platform only contained five probes and, collectively, the individual probe results were not informative for confidently calling a deletion. Thus, based on these findings, we speculate that NGS will not only be a useful technique for detecting additional genome-wide CNVs contributing to disease phenotypes, but also a preferable technology for precisely delineating CNVs at the terminal ends of chromosomes, with particular application to the analysis of all types of ring chromosomes. Further, based on similar principles, NGS technology may also have useful application for the diagnosis of unbalanced translocations with small subtelomeric duplications and deletions and, aid in defining more precise phenotypes associated with these structural re-arrangements.

The cytogenetic and molecular karyotypes defined provided a sound basis for exploring possible genotype to phenotype correlations in the patient studied. The 6p25.3 microdeletion syndrome is a known chromosome disease with well-described clinical features, consisting of developmental delay, mental retardation, language impairment, hearing loss, and ophthalmologic, cardiac, and craniofacial abnormalities [17–20]. Further, patients with unbalanced translocations involving deletion of the 6p 25.3 region, also display a similar phenotype [21]. These clinical features of all patients with interstitial deletions of 6p25.3 closely overlap with the clinical features of patients that carry a r6 chromosome [7] and strongly suggest that the main phenotypes displayed are primarily due to hemizygous expression of genes within the 6p25.3-pter interval. Therefore, in order to explore genotypic associations with congenital heart disease, mental retardation and craniofacial abnormalities observed in the six-year-old girl, we specifically analyzed the known function of the genes encoded within the subtelomeric 6p25.3 region (Table 1).

**Table 1:** Genotype to phenotype associations

Main clinical findings in the patient	Gene	References
Severe mental retardation, speech delay	FOXC1	[27–29]
	FOXF2	[30]
	GMDS	[31]
Congenital heart disease, ostium secundum defect, patent ductus arteriosus, pulmonary stenosis, left superior vena cava residues and coronary sinus distention	FOXC1	[24–27]
	FOXF2	[24, 25]
Teeth agenesis	FOXF2	[30]
Leukoma	IRF4	[37, 38]
Iridogoniodysgenesis anomaly and nystagmus	FOXC1	[27]
	FOXF2	[35]

The FOX family are a group of transcription factors characterized by a conserved 110 amino acid DNA binding domain that play an important synergistic role in embryonic development, tissue-specific gene expression, morphogenesis [22] as well as cardiovascular development [23]. Four members of the FOX gene family *FOXC1*, *FOXF2*, *FOXQ1* and *FOXCUT* are encoded by genes within the 6p25.3-pter interval (Fig. 4). RNA studies found that both FOXC1 and FOXF2 are highly expressed in the left ventricle [24, 25] whereas FOXQ1 was not expressed in the heart [22]. Loss of function studies comparing different models with normal or abnormal heart development have also shown that lower levels of several FOX proteins, including FOXC1, is strongly associated with the pathogenesis of heart failure. Further, studies of compound *FOXC1* and *FOXC2* mutant embryos identified a wide spectrum of cardiac abnormalities, including cardiac inflow and outflow dysplasia and abnormal formation of the epicardium [26]. Moreover, patients with *FOXC1* specific mutations often have identifiable cardiac abnormalities [27]. Based on these limited studies, we speculate that haplo-deficiency of *FOXC1* and, possibly *FOXF2*, may contribute to the complex heart abnormalities seen in the r6 patient.

The r6 patient also exhibited severe mental retardation and speech delay. Several studies suggest that reduced expression of genes *FOXC1*, *FOXF2* and *GMDS* which are located in the 6p25.3-pter deletion interval, affect normal brain and central nervous system (CNS) development [27–31]. In *FOXC1* null mice, significant cerebellum abnormalities were observed [27]. Brain MRI scans of mental retardation patients with known *FOXC1* gene deletions or missense mutations show a range of different cerebellum malformations including mega cisterna magna or cerebellar vermis hypoplasia [27, 28]. In other studies, FOXF2 was identified as a regulator of neural outgrowth through the modulation of nuclear active Akt [29] and FOXF1 was shown to be an important developmental regulator of the CNS [30]. Mutations in the *GMDS* gene which encodes an enzyme responsible for protein fucosylation, have also been found in a zebrafish model to cause defects in neuronal differentiation, axon branching and synapse formation [31]. In one key study, the severity of mental retardation in patients was found to be strongly associated with the size and position of *FOXC1* deletions and whether they extended further to encompass exons of the nearby *GMDS* gene [27]. Taken together, these collective studies point to haplo-deficiency of *FOXC1* and *GMDS* as the primary genes responsible for the severe mental disabilities exhibited by the r6 patient.

In regard to physical abnormalities of the patient (Fig. 1), craniofacial features were highly dysmorphic, with eye and tooth abnormalities particularly prominent. Based on several studies, *FOXC1* is believed to be the primary causative gene, although there is evidence that *FOXF2* and *IF4* genes also contribute to the phenotype. Human *FOXC1* heterozygous mutations are well known to affect eye development, causing a spectrum of ocular-associated anomalies including glaucoma and Axenfeld-Rieger syndrome [27, 32–34]. In mice, heterozygous mutations of *FOXF2* are associated with iridocorneal angle changes [35] and in one patient studied, partial iris hyperplasia was present even when the 6p deletion did not encompass the *FOXC1* gene [36]. Thus hemizygous expression of FOXC1 and FOXF2 may explain the corneal abnormalities, iridogoniodysgenesis and nystagmus observed in the patient. Further, the gene *IRF4* is important for human pigmentation of the hair skin and eyes [37, 38] and therefore loss of one copy of this gene may explain leukoma identified in the eyes of the patient. Lastly, during embryonic tooth development in rodents from the bud to differentiation stage, *FOXF2* mRNA was detected in the mesenchyme surrounding tooth germ cells, especially in the dental follicle adjacent to the outer enamel epithelium [30]. Thus, loss of one copy of the *FOXF2* gene may also be associated with the teeth agenesis exhibited by the patient. These studies suggest that while hemizygous expression of FOXC1 is probably the main cause of the craniofacial abnormalities in the patient, loss of one copy of the *FOXF2* and *IRF4* genes may have significantly exacerbated the severity of the facial abnormalities.

Based on a survey of the available literature, the evidence presented strongly points to haplodeficiency of the *FOXC1* gene as the major contributing factor to the overall phenotype of the patient and that hemizygous expression of *FOXF2*, *IRF4* and *GMDS* genes may further contribute to the phenotype. However, the phenotype of the patient was severer than that exhibited by other r6 patients reported with more extensive 6p and 6q deletions [7], in particular, growth retardation and mental development was severe. Within the 6q27 deletion interval there were 10 genes, with only *TBP* recognized as an OMIM disease gene. Trinucleotide repeat expansion of the *TBP* gene has been shown to cause the neurological disorder spinocerebellar ataxia 17 [39]. However, since patients with r6 chromosomes involving both 6p and 6q terminal deletions exhibit a very similar phenotype to patients with r6 chromosomes involving only 6p terminal deletions [7], we argue that the 6q terminal deletion carried by the child was unlikely to have significantly contributed to the severer phenotype. This leaves the finding of r6 mosaicism in this patient as the most likely explanation. In previous studies, evidence suggests that growth and developmental delay commonly seen in patients carrying autosomal ring chromosomes is due to mosaicism caused by instability of the ring chromosome [4, 40, 41]. On this basis, we speculate that low-level r6 mosaicism, probably originating early in neonatal development, has exacerbated the severity of the symptoms exhibited by this patient, which appear to be primarily caused by the 6p25.3-pter deletion event.

## Methods

### Study oversight

The research study was approved by the Medical Ethics Committee of the Second Affiliated Hospital of Harbin Medical University (Approval Number KY2016-154).

### Cytogenetic studies

Blood cell karyotyping was performed according to standard methods [42]. White blood cells were cultured for 72 h in PHA supplemented Serum Free Culture Medium (Guangzhou He Neng Bio Technology Co., Ltd). Skin puncture biopsies were taken from the abdominal skin, placed in PBS, cut into 0.5 mm^3^ pieces, transferred into a Dispase II solution and incubated overnight at 4 °C [43]. Epidermal fibroblasts were then cultured at 37 °C under 5 % CO_2_ and 90 % humidity for 12 days in PHA supplemented BIOAMFTM-3 media (Biological Industries Israel Beit Haemek Ltd). Following colcemid treatment, G banded metaphase chromosome spreads of blood and skin cells were prepared. For detection of mosaicism, a minimum of 50 cells were karyotyped.

### Chromosome microarray analysis

Array CGH was performed using 8 × 60 K commercial arrays (Agilent) according to the manufacturer’s recommended protocol. After DNA labeling, hybridization and washing, slides were scanned using an Agilent microarray scanner, and raw data extracted using Feature Extraction Software at the default CGH parameter settings. Putative copy number alteration intervals in each sample were identified using Agilent Genomic Workbench v6.5.0.18 software. Cy5/Cy3 ratios were converted into log2-transformed values and the Aberration Detection Method 2 algorithm applied at threshold 6.0 to identify CNVs, based on the following criteria: ≥ 5 probes per CNV interval and a minimum absolute average log2 ratio of ≥ 0.38 for the test region.

### Next generation sequencing

NGS was performed by using copy number variation sequencing (CNV-Seq) as previously described [16, 44]. DNA libraries were constructed and subjected to massively parallel sequencing on the Hi Seq2500 platform (Illumina) to generate 36 bp sequencing reads. High quality reads (2.8–3.2 million) were mapped to the hg19 reference genome [45], allocated to 20 kb sequencing bins and the mean CNV plotted for each chromosome [44].

### Consent

The parental guardians provided written informed consent on behalf of the child for publication of this Research Article and the accompanying image. A copy of the written consent form is available for review by the Editor-in-Chief of this journal.

## Conclusions

In this study, we demonstrate molecular utility of NGS as a high resolution technology for molecular characterization of subtelomeric deletions associated with a novel r6 chromosome. Based on the defined genotype, we attributed the severe mental retardation, congenital heart disease and craniofacial abnormalities observed in the patient largely to hemizygous expression of *FOXC1*, *FOXF2* and *GMDS* genes within the 6p25.3-pter interval, although circumstantial evidence suggested that in vivo instability of the r6 chromosome exacerbated the severity of the phenotype. With ethical approval and patient consent, further tissue studies are needed to better understand the genetic basis of the variable and complex phenotypes observed in rare patients with r6 chromosomes.

## Abbreviations

CMA: chromosome microarray analysis
CNV-Seq: copy number variation sequencing
NGS: next generation sequencing
r6: ring 6
